# Ochratoxin A-induced autophagy *in vitro* and *in vivo* promotes porcine circovirus type 2 replication

**DOI:** 10.1038/cddis.2017.303

**Published:** 2017-06-29

**Authors:** Gang Qian, Dandan Liu, Junfa Hu, Fang Gan, Lili Hou, Xingxiang Chen, Kehe Huang

**Affiliations:** 1 College of Veterinary Medicine, Nanjing Agricultural University, Nanjing 210095, Jiangsu Province, China; 2 Institute of Nutritional and Metabolic Disorders in Domestic Animals and Fowls, Nanjing Agricultural University, Nanjing 210095, Jiangsu Province, China

## Abstract

Ochratoxin A (OTA) is a mycotoxin produced by *Aspergillus* and *Penicillium*. Porcine circovirus type 2 (PCV2) is recognized as the causative agent of porcine circovirus-associated diseases. Recently, we reported that low doses of OTA promoted PCV2 replication *in vitro* and *in vivo*, but the underlying mechanism needed further investigation. The present studies further confirmed OTA-induced PCV2 replication promotion as measured by cap protein expression, viral titer, viral DNA copies and the number of infected cells. Our studies also showed that OTA induced autophagy in PK-15 cells, as assessed by the markedly increased expression of microtubule-associated protein 1 light chain 3 (LC3)-II, autophagy-related protein 5 (ATG5), and Beclin-1 and the accumulation of green fluorescent protein (GFP)-LC3 dots. OTA induced complete autophagic flux, which was detected by monitoring p62 degradation and LC3-II turnover using immunoblotting. Inhibition of autophagy by 3-methylademine (3-MA) and chloroquine (CQ) significantly attenuated OTA-induced PCV2 replication promotion. The observed phenomenon was further confirmed by the knock-down of ATG5 or Beclin-1 by specific siRNA. Further studies showed that *N*-acetyl-L-cysteine (NAC), an ROS scavenger could block autophagy induced by OTA, indicating that ROS may be involved in the regulation of OTA-induced autophagy. Furthermore, we observed significant increases in OTA concentrations in lung, spleen, kidney, liver and inguinal lymph nodes (ILN) and bronchial lymph nodes (BLN) of pigs fed 75 and 150 *μ*g/kg OTA compared with controls *in vivo*. Administration of 75 *μ*g/kg OTA significantly increased PCV2 replication and autophagy in the lung, spleen, kidney and BLN of pigs. Taken together, it could be concluded that OTA-induced autophagy *in vitro* and *in vivo* promotes PCV2 replication.

Porcine circovirus type 2 (PCV2) is a small, non-enveloped, single standard circular DNA virus belonging to the genus *Circovirus* of the *Circoviridae* family.^1^ PCV2 is the primary causative agent of several diseases that are collectively referred to as porcine circovirus-associated diseases (PCVAD). However, not all pigs infected with PCV2 develop PCVAD, and other factors, such as animal management, viral infections, immune stimulation and nutrition, have been suggested to be associated with the disease.^2^

Ochratoxins are a group of mycotoxins produced principally by ubiquitous strains of *Aspergillus* and *Penicillium*.^3, 4^ Ochratoxin A (OTA), the most prevalent ochratoxin.^5^ Owing to its natural origin, pervasiveness at low levels and chemical stability, OTA contamination persists throughout the feed supply and the food chain.^3, 6^ Our previous work suggests that OTA may be an important trigger of PCV2 replication and that the varying levels of OTA in pig feed may partially explain why the morbidity and severity of PCVAD vary among PCV2-infected pig farms.^7^ However, the underlying mechanism through which OTA promotes PCV2 replication requires further investigation.

Autophagy is a conserved catabolic intracellular process through which long-lived proteins and damaged organelles are degraded by the lysosomal machinery.^8, 9^ Autophagy not only plays an essential role in cellular homeostasis but also participates in numerous other physiological and pathological processes.^10^ Normally, autophagy acts as a defense mechanism against viral infection.^11, 12, 13^ Interestingly, several types of viruses exploited the autophagic processes to enhance their own replication.^13, 14, 15^ Few data involving autophagy, OTA and viral infection have been reported to date.

In the present study, we investigated the role of autophagy in OTA-promoted PCV2 replication *in vitro* and *in vivo*, thereby developing potential novel antiviral strategies against PCV2 infection.

## Results

### Cytotoxic effects of OTA on PK-15 cells

**Figure 1:**
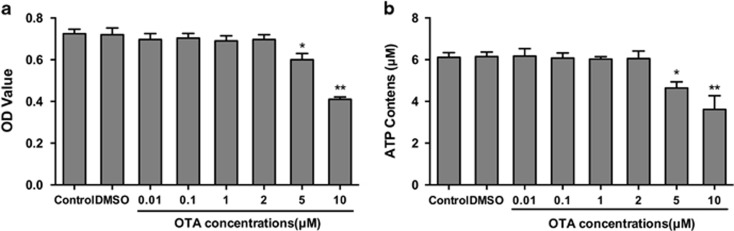
Effect of ochratoxin A (OTA) on the cell viability and ATP contents of PK-15 cells. PK-15 cells were seeded in 96-well plates at a density of 5 × 10^3^ cells/well or in 24-well plates at a density of 4 × 10^4^ cells/well and cultured in the presence of 0.01, 0.1, 1, 2, 5, or 10 *μ*M OTA for 48 h. (**a**) Cell viability was determined by MTT assay. (**b**) Intracelluar ATP concentrations were determinded using a luciferase-based ATP assay kit. The data are presented as means±S.E. of three independent experiments. Statistical significance compared with the control is indicated by *P<0.05 and **P<0.01

To determine whether the effect of OTA on PCV2 replication results from OTA induction of cell toxicity or alteration in the energy supply of PK-15 cells, we examined the effects of OTA on cell viability using the 3-(4,5-dimethyl-2-thiazolyl)-2,5-diphenyl-2-H-tetrazolium bromide (MTT) and ATP assays. As shown in Figure 1a, the viability of PK-15 cells was not affected by OTA up to a concentration of 2 *μ*M, but at concentrations of 5 and 10 *μ*M, OTA significantly lowered (*P<*0.05) the cell viability of PK-15 cells. A similar reduction in intracellular ATP levels after treatment of the cells with OTA was observed (Figure 1b). Thus, in subsequent experiments, OTA was used at concentrations between 0.01 and 2 *μ*M.

### OTA promotes PCV2 replication in PK-15 cells

**Figure 2:**
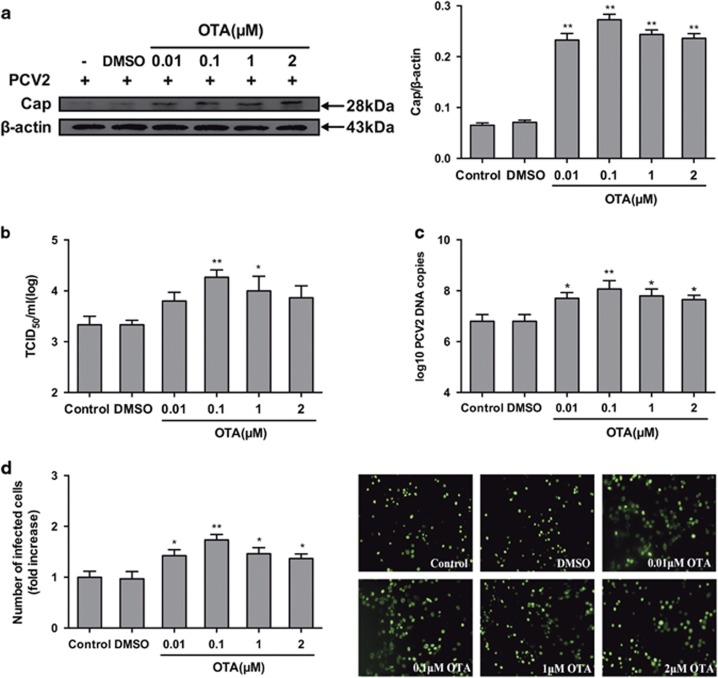
OTA promotes PCV2 replication in PK-15 cells. PK-15 cells were infected with PCV2 for 24 h and then treated with OTA at concentrations of 0.01, 0.1, 1, or 2 *μ*M for an additional 48 h. The cells were then collected and assayed for (**a**) PCV2 viral cap protein expression by western blotting, (**b**) viral titer by IFA, (**c**) PCV2 viral DNA copies by qRT-PCR, and (**d**) the number of infected cells by IFA, as described in the Materials and Methods section. The data are presented as means±S.E. of three independent experiments. Statistical significance compared with the control is indicated by *P<0.05 and **P<0.01

To determine the effect of OTA on PCV2 replication, PK-15 cells were infected with PCV2 for 24 h and then incubated with OTA at concentrations between 0.01 and 2 *μ*M for an additional 48 h. As shown in Figure 2, exposure of cells to the solvent control, dimethyl sulfoxide (DMSO), had no effect on PCV2 replication. However, treatment with 0.01, 0.1,1 or 2 *μ*M OTA significantly (*P<*0.05) increased the cap protein level (Figure 2a), viral titer (Figure 2b), PCV2 DNA copies (Figure 2c) and the number of PCV2-infected cells (Figure 2d) compared with the control group. The maximal effects were observed at 0.1 *μ*M OTA.

### OTA increases autophagosome formation in PK-15 cells

**Figure 3:**
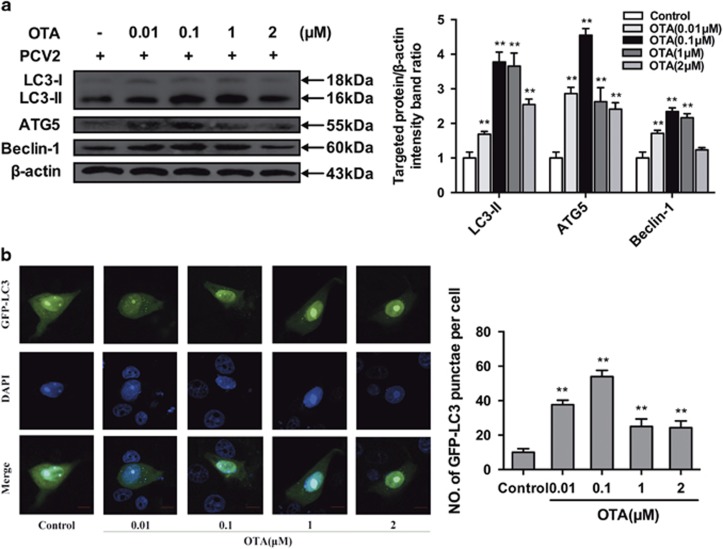
OTA induces autophagy in PK-15 cells. (**a**) PK-15 cells were inoculated with PCV2 for 24 h, OTA was then added at concentrations of 0.01, 0.1, 1, or 2*μ*M, and incubation was continued for an additional 48 h. After collecting the cells, the expression of LC3, ATG5, Beclin-1 and *β*-actin (loading control) was analyzed by immunoblotting with specific antibodies as described in Materials and Methods. The data are presented as means±S.E. of three independent experiments. Statistical significance compared with the control is indicated by *P<0.05 and **P<0.01. (**b**) PK-15 cells were first transfected with the GFP-LC3 plasmid. After 12 h, the cells were inoculated with PCV2 for 24 h, then, OTA was added at a concentration of 0.01, 0.1, 1, or 2 *μ*M, incubation was continued for 48 h, and the fluorescence signal was visualized by confocal immunofluorescence microscopy. Scale bar: 10 *μ*m. The average number of LC3 puncta in each cell was determined from at least 100 cells in each group. The data are presented as means±S.E. of three independent experiments. Statistical significance compared with the control is indicated by *P<0.05 and **P<0.01

To determine whether OTA induces autophagy in PCV2-infected PK-15 cells, we examined the levels of autophagy marker proteins using Western blotting. LC3, which is necessary for the formation and maturation of the autophagosome, is a hallmark for assessing autophagy.^16, 17^ Our results showed that treatment with OTA led to a significant upregulation of LC3-II expression (Figure 3a). We next examined the expression of ATG5, an essential protein that is associated with the formation of the autophagosomes membranes.^18^ OTA-treated cells displayed significantly increased levels of ATG5 relative to control cells (Figure 3a). We further measured the levels of Beclin-1, a protein involved in the early steps of the autophagy pathway.^19, 20^ Similar results were also observed in that OTA treatment induced the overexpression of Beclin-1 relative to control cells (Figure 3a). The maximal effects of OTA on the expression of autophagic markers were observed at an OTA concentration of 0.1 *μ*M.

To further analyze whether the autophagy machinery was triggered by OTA treatment, PK-15 cells were transfected with green fluorescent protein-microtubule-associated protein 1 light-chain 3 (GFP-LC3), a specific marker of autophagic vesicles and autophagic activity.^21, 22^ As shown in Figure 3b, OTA treatment significantly increased the number of GFP-LC3 puncta compared with the control group, with the maximal effect observed at an OTA concentration of 0.1 *μ*M. These findings indicate that OTA induces autophagy in PCV2-infected PK-15 cells.

### OTA treatment increases autophagic flux

**Figure 4:**
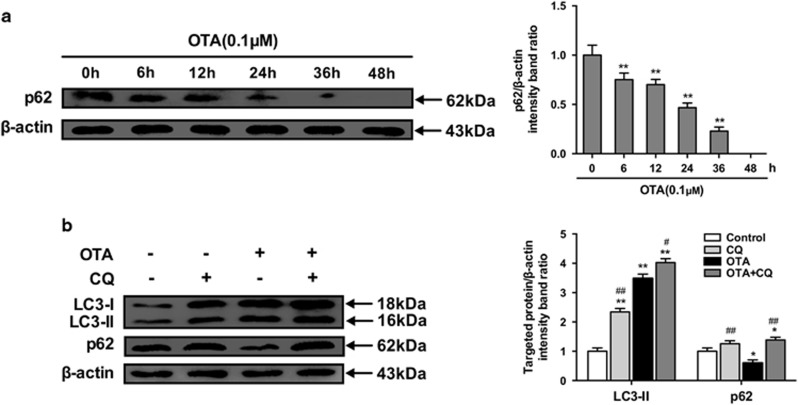
OTA treatment enhances autophagic flux. (**a**) PK-15 cells were inoculated with PCV2 for 24 h and then inculated with OTA (0.1 *μ*M). At the indicated times after inoculation, the cells were collected, and the expression of p62 and *β*-actin (loading control) was analyzed by immunoblotting with specific antibodies as described in Materials and Methods. The data are presented as means±S.E. of three independent experiments. Statistical significance compared with the control is indicated by *P<0.05 and **P<0.01. (**b**) PCV2-infected PK-15 cells were incubated with OTA (0.1 *μ*M), CQ or with OTA and CQ for 48 h. After collecting the cells, the expression of LC3, p62 and *β*-actin (loading control) was analyzed by immunoblotting with specific antibodies as described in Materials and Methods. The data are presented as the mean±S.E. of three independent experiments. Statistical significance compared with the control is indicated by *P<0.05 and **P<0.01. Statistical significance compared with OTA is indicated by ^#^*P*<0.05 and ^##^*P*<0.01

To determine whether a complete autophagic flux is occurred by OTA treatment, we investigated the degradation of p62.^22, 23, 24^ As shown in Figure 4a, the level of p62 protein in PK-15 cells decreased significantly with time during OTA treatment.

Since enhanced autophagosome accumulation could be caused either by increased autophagosome formation or by decreased autophagosome turnover,^25^ the levels of LC3-II and p62 were measured in the presence of the lysosomal protease inhibitor CQ.^26^ As demonstrated in Figure 4b, CQ treatment significantly increased the levels of LC3-II and p62 in OTA-treated PK-15 cells, suggesting that OTA treatment enhanced autophagic flux in the cells.

### Pharmacological inhibition of autophagy reduces OTA-promoted PCV2 replication in PK-15 cells

**Figure 5:**
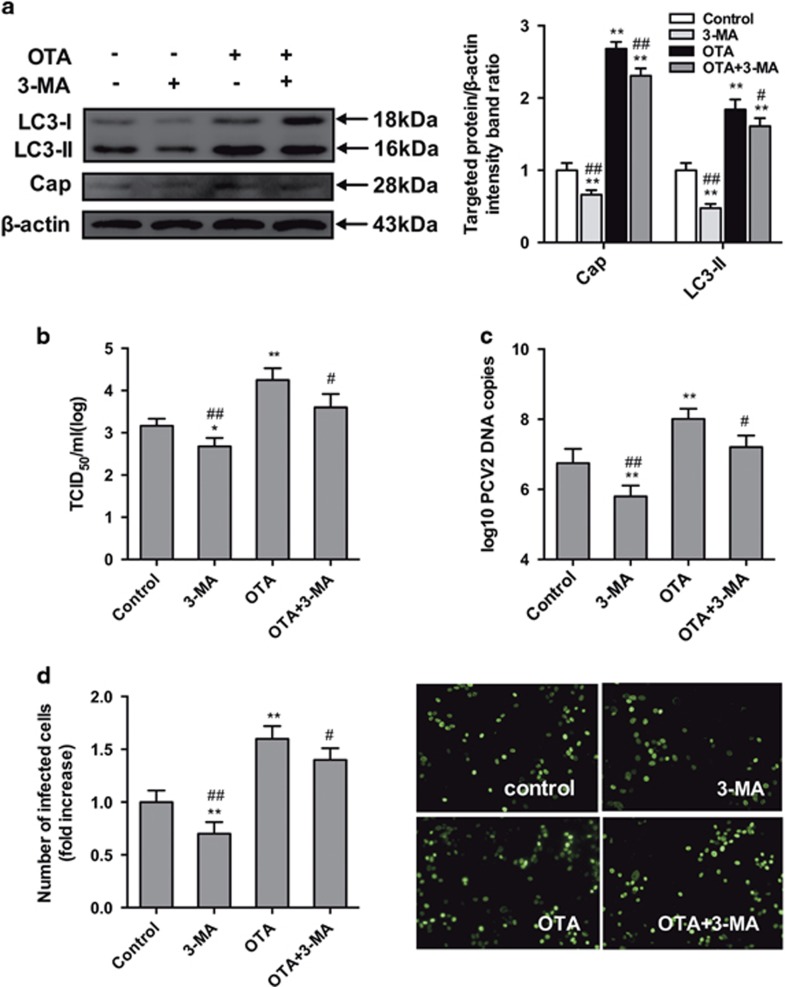
Inhibition of autophagy with 3-MA reverses PCV2 replication induced by OTA in PK-15 cells. PCV2-infected cells were incubated with OTA (0.1 *μ*M) with or without 3-MA (5 mM). The cells were then assayed for (**a**,**b**) expression levels of LC3, Cap and *β*-actin (loading control), (**c**) PCV2 viral titers, (**d**) PCV2 viral DNA copies and (**e**) the number of infected cells, as described in Materials and methods. The data are presented as means±S.E. of three independent experiments. Statistical significance compared with the control is indicated by *P<0.05 and **P<0.01. Statistical significance compared with OTA is indicated by ^#^*P*<0.05 and ^##^*P*<0.01

To evaluate the role of autophagy in OTA-promoted PCV2 replication in PK-15 cells, the effect of 3-MA, which inhibits autophagy by blocking the formation of autophagosomes,^27, 28^ on OTA-promoted PCV2 replication was examined. Figure 5 shows that treatment with 3-MA reduced the level of cap protein (Figure 5a), viral titers (Figure 5b), PCV2 DNA copies (Figure 5c) and the number of infected cells (Figure 5d), compared with the control group and that is attenuated the promotion of PCV2 replication by 0.1 *μ*M OTA (Figures 5a–d).

**Figure 6:**
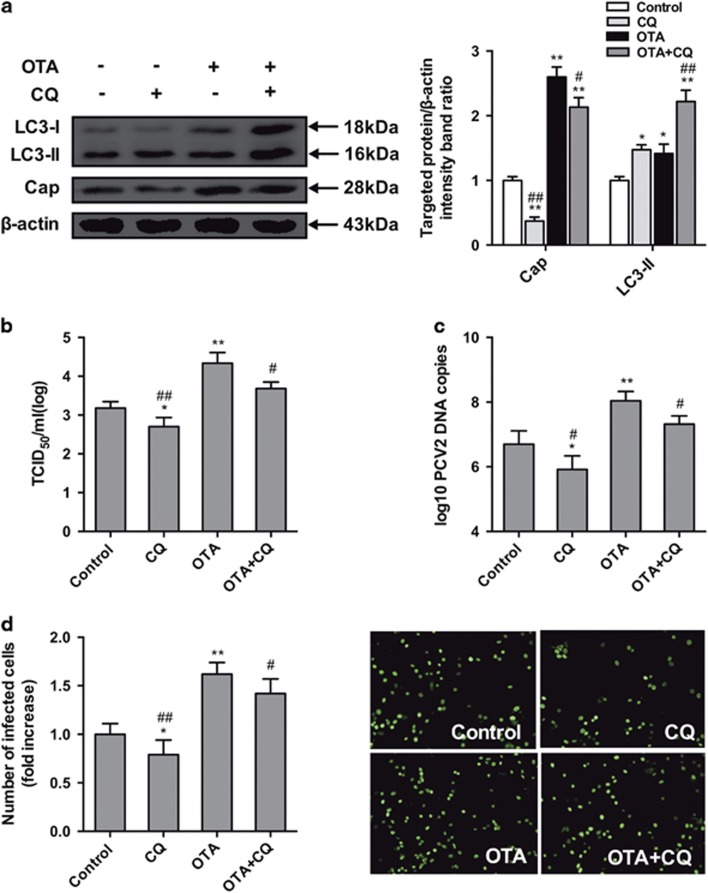
Inhibition of autophagy by CQ reverses PCV2 replication promotion induced by OTA in PK-15 cells. PCV2-infected cells were incubated with OTA (0.1 *μ*M) with or without CQ (5 *μ*M). Cells were assayed for (**a**,**b**) expression levels of LC3, Cap and *β*-actin (loading control), (**c**)PCV2 viral titers, (**d**) PCV2 viral DNA copies and (**e**) the number of infected cells as described in Materials and Methods. The data are presented as means±S.E. of three independent experiments. Statistical significance compared with the control is indicated by *P<0.05 and **P<0.01. Statistical significance compared with OTA is indicated by ^#^*P*<0.05 and ^##^*P*<0.01

Similarly, CQ, an inhibitor of autophagic flux, was utilized to inhibit the late stage of autophagy.^29^ As demonstrated in Figure 6, CQ treatment significantly decreased the expression of cap protein (Figure 6a), viral titers (Figure 6b), PCV2 DNA copies (Figure 6c) and the number of infected cells (Figure 6d) compared with the control group, it also decreased the promotion of PCV2 replication by 0.1 *μ*M OTA (Figures 6b–e).

The results of these pharmacological experiments demonstrated that autophagy has a positive effect on OTA-promoted PCV2 replication.

### Knockdown of autophagy-related genes (ATGs) reduces OTA-promoted PCV2 replication

**Figure 7:**
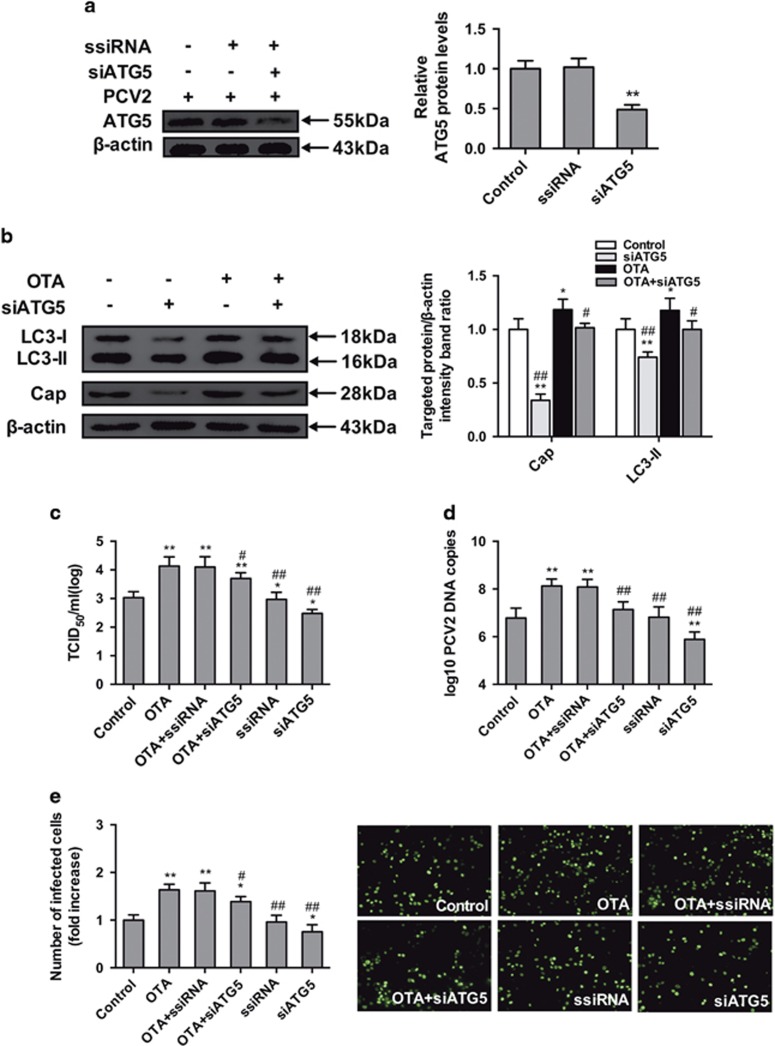
Inhibition of autophagy with siATG5 reverses the PCV2 replication promotion induced by OTA in PK-15 cells. PCV2-infected cells were incubated with or without ATG5 siRNA. The cells were then assayed for the expression levels of (**a**) ATG5 and *β*-actin (loading control). The data are presented as means±S.E. of three independent experiments. Statistical significance compared with the control is indicated by *P<0.05 and **P<0.01. PCV2-infected cells were transfected with ATG5-specific siRNA or a control siRNA and incubated with OTA (0.1 *μ*M) for 48 h. Cells were assayed for (**b**) expression levels of LC3, Cap and *β*-actin (loading control), (**c**) PCV2 viral titers, (**d**) PCV2 viral DNA copies, and (**e**) the number of infected cells, as described in Materials and Methods. The data are presented as means±S.E. of three independent experiments. Statistical significance compared with the control is indicated by *P<0.05 and **P<0.01. Statistical significance compared with OTA is indicated by ^#^*P*<0.05 and ^##^*P*<0.01

**Figure 8:**
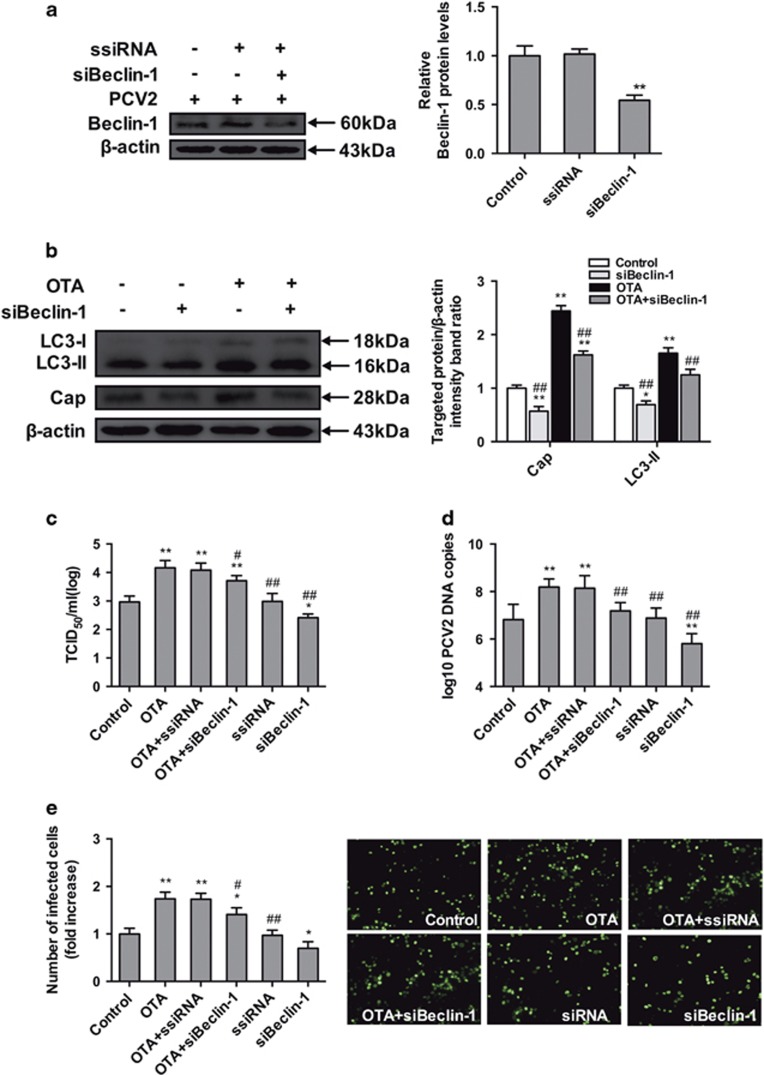
Inhibition of autophagy with siBeclin-1 reverses the PCV2 replication promotion induced by OTA in PK-15 cells. PCV2-infected cells were incubated with or without Beclin-1 siRNA. Cell were assayed for expression levels of (**a**) Beclin-1 and *β*-actin (loading control). The data are presented as means±S.E. of three independent experiments. Statistical significance compared with the control is indicated by *P<0.05 and**P<0.01. PCV2-infected cells were transfected with Beclin-1-specific siRNA or a control siRNA and incubated with OTA (0.1 *μ*M) for 48 h. Cells were assayed for (**b**) expression levels of LC3, Cap and *β*-actin (loading control), (**c**) PCV2 viral titers, (**d**) PCV2 viral DNA copies, and (**e**) the number of infected cells as described in Materials and Methods. The data are presented as means±S.E. of three independent experiments. Statistical significance compared with the control is indicated by *P<0.05 and **P<0.01. Statistical significance compared with OTA is indicated by ^#^*P*<0.05 and ^##^*P*<0.01

To exclude any non-specific effects in our pharmacological experiments, we further examined the effect of reducing the levels of the intracellular autophagy proteins ATG5 and Beclin-1 on the replication of PCV2 promoted by OTA, using target-specific RNA interference. As shown in Figures 7a and 8a, PK-15 cells transfected with siATG5 and siBeclin-1 displayed significantly decreased expression levels of the ATG5 and Beclin-1 proteins compared with cells transfected with ssiRNA and with the control group. As shown in Figures 7 and 8, significant reduction in viral cap protein expression (Figures 7b and 8b), viral titers (Figures 7c and 8c), PCV2 DNA copies (Figures 7d and 8d) and the number of infected cells (Figures 7e and 8e) in cultures of PK-15 cells transfected with siATG5 or siBeclin-1 without or with treatment of OTA were observed. These results are in agreement with the results of previous pharmacological studies. Taken together, these results further support the idea that autophagy is required for OTA-promoted PCV2 replication.

### OTA induces autophagy via ROS induction

**Figure 9:**
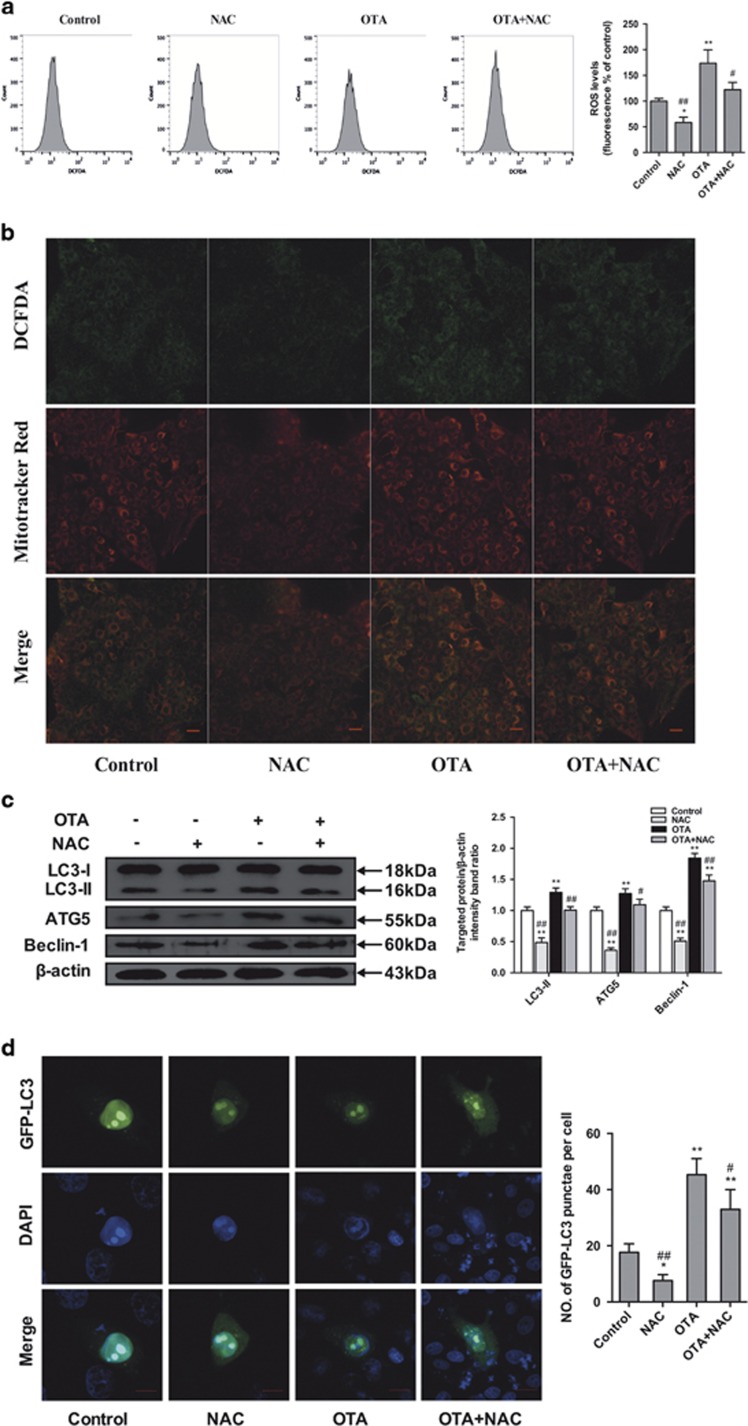
Effects of OTA and/or NAC on oxidative stress and autophagy in PCV2-infected PK-15 cells. PK-15 cells were inoculated with PCV2 for 24 h and then inculated with OTA (0.1 *μ*M), NAC (5 mM), or OTA and NAC together for an additional 48 h. (**a**) The cells were then incubated with DCFH-DA (10 *μ*M) at 37 °C for 30 min. The level of ROS was determined by flow cytometry. The level of intracellular ROS, which was indicated by an increase in the fluorescence intensity of the cells, was calculated as the percentage of that of the control cells. (**b**) Representative flourescent staining showing ROS visualized by DCFH-DA fluoreseence (green), and mitochondria labeled by MitoTracker Red CMXRos (red) in PK-15 cells, yellow color (green plus red) indicates the colocalization of ROS and mitochondria. Scale bar: 10 *μ*m. (**c**) The cells were collected, and the expression levels of LC3, ATG5, Beclin-1 and *β*-actin (loading control) were analyzed by immunoblotting with specific antibodies as described in Materials and Methods. (**d**) PK-15 cells were first transfected with the GFP-LC3 plasmid. After 24 h, the cells were inoculated with PCV2 for 24 h and then incubated with OTA (0.1 *μ*M), NAC (5 mM), or OTA and NAC together for an additional 48 h. The fluorescence signals were visualized by confocal immunofluorescence microscopy. Scale bar: 10 *μ*m. The average number of LC3 puncta in each cell was determined from at least 100 cells in each group. The data are presented as means±S.E. of three independent experiments. Statistical significance compared with the control is indicated by *P<0.05 and **P<0.01. Statistical significance compared with OTA is indicated by ^#^*P*<0.05 and ^##^*P*<0.01

To assess the role of ROS in OTA-induced autophagy, we first determined whether ROS accumulate after the treatment of cells with OTA and whether the commonly used ROS inhibitor NAC inhibits ROS production. FACS analysis revealed an increase in the mean DCFH-DA fluorescence after the treatment of the cells with OTA (Figure 9a). Similar results were observed in cells stained with DCFH-DA and examined by confocal microscopy. As shown in Figure 9b, DCFH-DA fluorescence significantly increased after OTA treatment, whereas NAC could significantly decrease OTA-induced ROS production (Figures 9a and b). As shown in Figure 9b, the majority of the green fluorescence (DCFH-DA staining) was localized in the mitochondria after OTA treatment, indicating that mitochondria are the major sources of intracellular ROS. To verify that OTA-induced autophagy is associated with ROS induction, the level of autophagy was determined in the presence or absence of NAC. As shown in Figures 9c and d, OTA significantly increased (*P<*0.05) the expression levels of LC3-II, ATG5 and Beclin-1 and the formation of GFP-LC3 puncta. However, pretreatment with NAC markedly attenuated the induction of autophagy by OTA. These results indicate that OTA-induced autophagy was mediated by ROS induction.

### Alteration of autophagy does not affect cell viability

**Figure 10:**
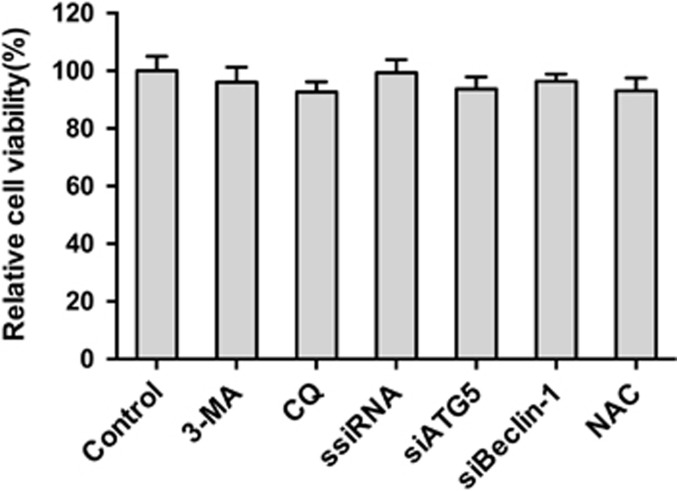
Pharmacological or siRNA alterations of autophagy and NAC does not affect cell viability. Cell viability was determined by MTT assay after treatment of PK-15 cells with 3-MA or CQ or NAC for 48 h or after transfection with ssiRNA, siATG5 or siBeclin-1 for 48 h. The data are presented as means±S.E. of three independent experiments

To assess whether the pharmacological alteration of autophagy with 3-MA and CQ or transfection with siRNA or NAC affected PCV2 replication by changing the cell viability, the effects of our pharmacological treatments or siRNA transfection or NAC on PK-15 cell viability were analyzed using the MTT assay. Statistical analyses showed no significant differences (*P*>0.05) on the viability of cells treated with agents or siRNA transfection (Figure 10).

### OTA treatment *in vivo*

**Figure 11:**
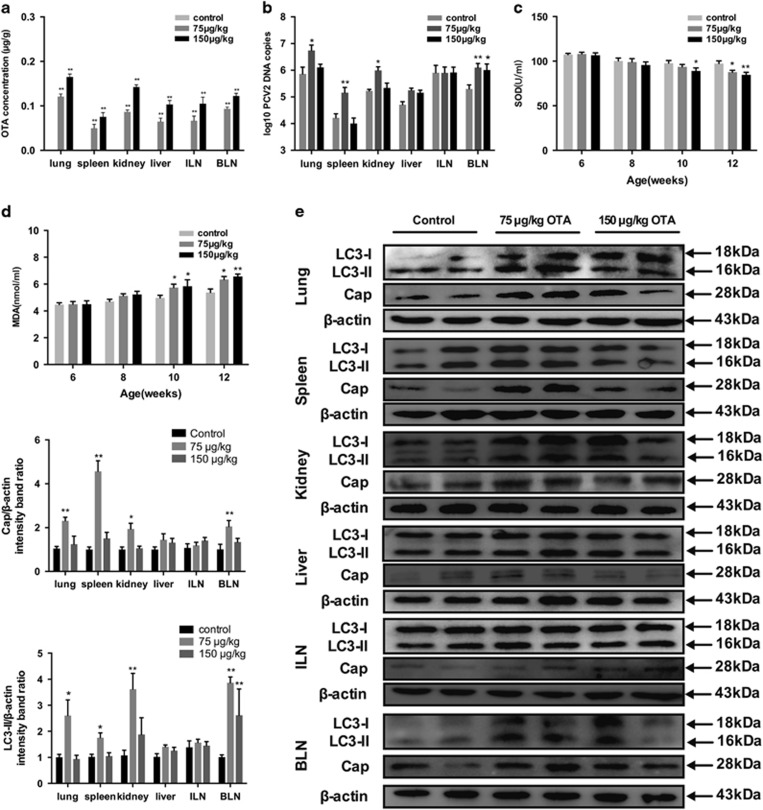
OTA, SOD and MDA concentrations, PCV2 DNA copies and western blotting of LC3 and cap in tissues of pigs fed a basal diet or a basal diet containing OTA at 75 and 150 *μ*g/kg. (**a**) OTA concentrations in lung, spleen, kidney, liver, ILN, and BLN of pigs fed the basal diet (control) or the basal diet with added OTA at 75 and 150 *μ*g/kg. (**b**,**c**) Levels of (**b**) SOD and (**c**) MDA in serum from pigs fed the basal diet (control) or the basal diet containing OTA at 75 or 150 *μ*g/kg. (**d**) PCV2 DNA copies in lung, spleen, kidney, liver, ILN, and BLN of the same pigs were assessed by real-time PCR. (**e**) The expression levels of LC3 and cap in lung, spleen, kidney, liver, ILN, and BLN of the same pigs were measured by western blotting. The data are presented as means±S.E. of three independent experiments. Statistical significance compared with the control is indicated by *P<0.05 and **P<0.01

We further evaluated the effects of OTA on PCV2 infection in an animal model and determined whether autophagy can also be induced *in vivo*. As Figure 11a shows, no OTA was detected in the tissues from the control group, whereas OTA concentrations were significantly increased in the tissues of pigs fed an OTA-contaminating diet. The levels of OTA vary from 0.04 to 0.12 *μ*g/g in the tissues of the animals in the 75 *μ*g/kg OTA group and from 0.08 to 0.16 *μ*g/g in the tissues of the animals in the 150 *μ*g/kg OTA group. These concentrations are similar to concentrations of OTA that promoted PCV2 replication in the *in vitro* experiment.

Next, we determined the PCV2 viral loads in the tissues of the three groups of pigs using qRT-PCR. Figure 11b shows that, compared with the pigs in the control group, significant increases in PCV2 DNA copies were found in the lungs, spleens, and kidneys of the pigs treated with 75 *μ*g/kg OTA and in the bronchial lymph nodes (BLN) of the pigs treated with 75 and 150 *μ*g/kg OTA. We also analyzed the PCV2 viral cap protein levels in the tissues of all the animals by Western blotting. We observed that the lungs, spleens, kidneys, and BLN of the pigs treated with 75 *μ*g/kg OTA contained significantly higher expression levels of cap protein (Figure 11e). These *in vivo* results are consistent with the *in vitro* data showing that relatively low doses of OTA could promote PCV2 replication.

Serum SOD activity and serum MDA content were measured following the treatment of the animals with OTA. As shown in Figures 11c and d, following the feeding of OTA-containing diets, significant reductions in SOD activities compared with those in the control group were observed in the 75 *μ*g/kg OTA group on day 42 and in the 150 *μ*g/kg OTA group on days 28 and 42, whereas slight reductions in SOD activity were observed on days 14 and 28 in the 75 *μ*g/kg OTA group and on day 14 in the 150 *μ*g/kg OTA group (Figure 11c). In addition, significant increases in MDA levels were observed in both OTA-fed groups on days 28 and 42, whereas slight increases were observed on day 14 (Figure 11d).

We also measured the autophagy levels in the tissues of OTA-fed animals by measuring the LC3-II/*β*-actin ratio in the tissues. Figure 11e shows that the LC3-II/*β*-actin ratio increased significantly in the lungs, spleens, and kidneys of pigs treated with 75 *μ*g/kg OTA and in the BLN of pigs treated with 75 and 150 *μ*g/kg OTA, compared with pigs that received basal diet. The LC3-II/*β*-actin ratios in the examined tissues were generally consistent with the viral infection levels in those tissues.

Overall, these results indicated that low doses of OTA could promote PCV2 replication and induce oxidative stress, and active autophagy *in vivo*.

## Discussion

The presence of mycotoxins derived from naturally occurring fungal metabolites in feed and foodstuffs of humans and animals is unavoidable. Owing to their widespread presence in the environment, mycotoxins often co-exist with other infectious agents such as viruses, bacteria and other pathogens and may increase the susceptibility to these infectious agents. OTA is a natural contaminant of farm animal feeds worldwide and poses a potential threat to animal production.^30^ Among farmed animals, pigs are particularly sensitive to OTA and are frequently exposed to this mycotoxin. PCV2 is an emerging viral disease that is a major concern for pig health and has significant economic impact on the swine industry, but the morbidity and severity of PCVAD vary among pig farms.^31, 32^ Numerous cofactors have been shown to contribute to the development of PCVAD. Our previous work suggests that OTA may be an important trigger of PCV2 replication and that the varying levels of OTA in pig feed may partly explain the difference in the morbidity and severity of PCVAD vary among pig farms.^7^ However, the underlying mechanisms of OTA-promoted PCV2 replication require further investigation.

Autophagy is an evolutionarily ancient pathway that plays essential roles in host defenses against viral infection and cell survival;^33, 34^ for instance, it is involved in cellular defenses against herpes simplex virus and mosaic virus infections.^35, 36^ In contrast, many viruses exploit the autophagy machinery to enhance their own replication.^11, 13, 14^ Mycotoxins have generally not been associated with autophagy. Recently, however, mycotoxins such as patulin, zearalenone and OTA have been linked to autophagy.^34, 37, 38^ Based on recent findings concerning the interplay between mycotoxin, autophagy and viral infection, an autophagic mechanism appears to be involved in OTA-promoted PCV2 replication. In this study, we determined the role of autophagy in OTA-promoted PCV2 replication. Our results reveal a novel mechanism by which ROS-mediated autophagy induced by OTA contributes to PCV2 infection both *in vitro* and *in vivo*.

In the present study, the viability of PK-15 cells was significantly decreased by OTA at concentrations above 2 *μ*M, consistent with previous studies.^39, 40^ As shown in Figure 1, no cell cytotoxicity or change in ATP contents was observed at OTA concentrations between 0.01 and 2 *μ*M; however, PCV2 replication was significantly promoted by OTA (Figure 2), and maximal promotion of PCV2 replication was observed at 0.1 *μ*M OTA. The results further confirmed that low doses of OTA promote PCV2 replication in PK-15 cells^7^ and that the promotion of PCV2 replication observed in these experiments was due to OTA exposure.

In the present study, we provide evidence that non-toxic concentrations of OTA induce autophagy in PCV2-infected PK-15 cells through increased expression of LC3-II, ATG5, and Beclin-1. Consistently, a large number of cytoplasmic autophagosomes were observed by confocal fluorescence microscopy in OTA-treated PK-15 cells, further confirming that autophagy was induced by OTA. However, previous studies have demonstrated that the induction of autophagy involves not merely an increase in the formation of autophagosomes, but also an increase in autophagic flux.^26^ P62 degradation is a method that is widely used to assess autophagic flux.^23, 41^ In our study, we found that the p62 expression level was significantly decreased in PCV2-infected PK-15 cells after the treatment with OTA over a prolonged period of time. We also measured the levels of LC3-II and p62 in the presence or absence of lysosome inhibitor CQ. Treatment with CQ could lead to a pH shift in the, thus inhibiting lysosomal acidification. Figure 4b shows that treatment of PK-15 cells with OTA decreased the p62 expression level in the cells and that treatment with CQ increased the amounts of LC3-II and p62 in OTA-treated cells, indicating that the accumulation of autophagosomes in OTA treated PCV2-infected PK-15 cells was due to the increased autophagy flux.

Previous studies have shown that many viruses, including poliovirus, coxsackievirus B4, hepatitis C virus, dengue virus, and PCV2, utilize components of the autophagic pathway to facilitate their replication.^14, 15, 42, 43, 44^ However, the role of autophagy in OTA-promoted PCV2 replication has not been previously investigated.

To determine the role of autophagy in OTA-promoted PCV2 replication, PK-15 cells were treated with 3-MA and CQ, which are pharmacological inhibitors of autophagy.^45, 46^ These compounds inhibited PCV2 replication and reduced the replication of PCV2 promoted by OTA, consistent with previous studies showing that PCV2 replication was significantly reduced by 3-MA^14^ and that NDV replication was significantly reduced by CQ.^47^

To further confirm the pharmacological data and exclude any non-specific effects of these pharmacological agents used in our experiments, we used target-specific RNA interference to examine the effect of reducing the levels of essential autophagy proteins ATG5 and Beclin-1 on the replication of PCV2 promoted by OTA. The ATG5 protein is necessary for the formation and maturation of autophagosome,^48, 49^ whereas Beclin-1 is an essential autophagy protein that regulates the initiation of autophagy and autophagosome-lysosome fusion by interacting with two protein complexes. Both proteins are necessary for autophagy to occur.^50, 51^ Depletion of ATG5 and Beclin-1 proteins dramatically suppressed the formation of OTA-induced autophagosomes and attenuated the promotion of PCV2 induced by OTA. The results are consistent with those of previous studies in which it was demonstrated that suppression of ATG5 and Beclin-1 with siRNAs reduced the replication of dengue virus, influenza A virus, and PCV2.^13, 15, 52^

Accumulating evidence suggests that ROS and oxidative stress play a critical role in viral infection.^53, 54^ We previously found that oxidative stress can enhance PCV2 infection and that OTA can induce oxidative stress and thereby promote PCV2 replication.^7, 55^ Recently, an increasing body of research has indicated that ROS play an important role in controlling autophagy.^56, 57^ Oxidative stress has also been implicated in the mechanisms of OTA-induced cytotoxicity. Previous studies have explored OTA-induced oxidative stress in a variety of cell lines.^58, 59^ Therefore, we proposed that autophagy induced by OTA is mediated by the induction of ROS. Indeed, we observed significantly increased levels of ROS after OTA treatment of cells. In addition, we observed that the expression levels of LC3-II, ATG5 and Beclin-1 and OTA-induced GFP-LC3 puncta formation were markedly attenuated when ROS formation was inhibited by NAC. Collectively, our data indicate that OTA triggers oxidative stress and thereby induces autophagy.

During the past few years, numerous *in vitro* studies have demonstrated the critical role of autophagy in virus infection. However, few studies have been conducted using *in vivo* models. To corroborate our *in vitro* results, we evaluated the effects of OTA on PCV2 infection in an animal model and determined whether oxidative stress and autophagy can also be induced *in vivo*. In the *in vivo* experiment, pigs were fed diets containing 75 or 150 *μ*g/kg OTA. After such feeding, we observed OTA accumulation in various tissues. The highest concentration of OTA was found in lung tissue, followed by kidney, BLN, liver, inguinal lymph nodes (ILN), and spleen. This is apparently inconsistent with the fact that kidney is generally considered as the target organ for OTA toxicity.^60, 61^ However, similar results have also been reported in rats treated with low doses of OTA.^62^ In our experiments, the ranges of OTA concentrations in tissues were 0.05–0.11 *μ*g/g and 0.40–0.69 *μ*g/kg in the animals in the 75 *μ*g/kg OTA and 150 *μ*g/kg OTA treatment groups, respectively. The observed levels of OTA in the tissues of the treated animals are consistent with the concentrations of OTA that promoted PCV2 replication in PK-15 cells *in vitro*. Our data further showed that treatment of pigs with 75 *μ*g/kg OTA significantly increased PCV2 DNA copies and viral cap protein expression levels in various tissues, including lung, spleen, kidney, and BLN of pigs, a finding that is consistent with our *in vitro* results showing that relatively low doses of OTA can promote PCV2 replication.

In the present study, the oxidative stress status of pigs was examined. Increased levels of MDA in the serum and decreased serum SOD activities were observed following the treatment of the animals with OTA (Figures 11c and d). In addition, lapidated LC3-II was detected by western blotting in the tissues, and enhanced levels of LC3-II were observed in the lungs, spleens, and kidneys of pigs fed 75 *μ*g/kg OTA and in the BLN of pigs fed with 75 or 150 *μ*g/kg OTA. These results agree to some extent with the viral infectious levels measured in these animals.

Overall, these *in vitro* results reported here indicate that OTA-induced autophagy is mediated by oxidative stress and that it affects PCV2 replication. The increased levels of oxidative stress and LC3-II observed in the tissues of OTA-treated, PCV2-infected pigs indicate that oxidative stress and autophagy also occur in response to OTA treatment *in vivo*. These findings increase our knowledge of the pathogenesis of PCV2 and provide new insight into possible methods for the control of PCVAD.

## Materials and Methods

### Reagents and antibodies

MTT, 3-MA, CQ, anti-LC3 and HRP-conjugated anti-mouse and anti-rabbit antibodies were purchased from Sigma-Aldrich (St. Louis, MO, USA). NAC, 4′,6-diamidino-2-phenylindole and DCFH-DA were purchased from Beyotime Institute of Biotechnology (Haimen, Jiangsu Province, China). Anti-p62, anti-ATG5, anti-Beclin-1, and anti-*β*-actin antibodies were purchased from Santa Cruz Biotechnology (Santa Cruz, CA, USA). X-tremeGENE siRNA transfection regent was from Roche (Basel, Switzerland). MitoTracker Red was obtained from Invitrogen (Carlsbad, CA, USA).

### Cells, virus, and plasmids

PK-15 cells were provided by the China Institute of Veterinary Drug Control (China) and were free of PCV. PK-15 cells. PK-15 cells were maintained in Dulbecco’s minimal Eagle’s medium (DMEM; Invitrogen, Carlsbad, CA, USA) containing 8% fetal bovine serum (FBS; Gibco, Grand Island, NY, USA) and 1% antibiotics. The cells were cultured at 37 °C in a humidified atmosphere containing 5% CO_2_.

The wild-type PCV2 (PCV2NJ2002) used in the experiments was isolated originally from a kidney tissue sample of a pig with naturally occurring PMWS. The determination of PCV type was performed by sequencing (Invitrogen, Carlsbad, CA, USA). Stocks of PCV2 were generated from PK-15 cells infected with PCV2 as previously described.^55^

To construct pEGFP-LC3B, the LC3B gene was amplified from PK-15 cells using primers (LC3F: 5′-GAAGATCTGGGCTGAGGAGACACAAGAG-3′ LC3R: 5′-CGGAATTCTCTCAGTTGGTAACATCCCTTT-3′) that were designed based on the sequence of LC3B (GenBank No. NM 001190290.1). The gene was then cloned into pEGFP-C1 to express LC3B fused with the EGFP protein at its N-terminus.

### Virus infection and cell treatment

According to the requirements of individual experiments, PK-15 cells were infected with PCV2 at a multiplicity of infection of 1 at 37 °C. Following a 1-h absorption period, the cell monolayers were rinsed with sterile phosphate-buffered saline, pH 7.2 (PBS) to remove unattached viruses and then incubated in the presence of fresh medium at 37 °C for 24 h. Then the cells were then treated with various concentrations of OTA and incubated for an additional 48 h. For autophagy studies, PCV2-infected PK-15 cells were pretreated with 3-MA or CQ for 4 h at 37 °C. The cells were then treated as described above and further incubated in fresh medium in the absence or presence of 3-MA and CQ at the same concentrations as used in the pretreatments or in medium containing a corresponding amount of the solvent DMSO(control), unless otherwise specified in the figure legends.

### Cell viability assays

Cell viability was measured using the MTT assay according to the manufacturer’s instructions. Briefly, PK-15 cells were cultured in 96-well plates at a density of 5 × 10^3^ cells/well for 24 h and then subjected to various treatments for the specified periods. Following treatment, the culture medium was supplemented with 15 *μ*l of MTT (5 mg/ml) for 4 h at 37 °C. The supernatants were carefully aspirated and replaced with 150 *μ*l DMSO to dissolve the precipitate. Absorbance was measured in a microplate reader spectrophotometer at a wavelength of 570 nm. All tests were performed three times.

### Detection of cellular ATP levels

Cellular ATP levels were measured using a firefly luciferase-based ATP assay kit (Beyotime, Jiangsu Province, China) according to the manufacturer’s instructions. Briefly, PK-15 cells were cultured in 24-well plates at a density of 4 × 10^4^ cells/well for 24 h. After treatment with various concentrations of OTA for 48 h, the cells were schizolyzed and centrifuged at 12 000 × *g* for 5 min. Then, 50 *μ*l of each supernatant and 50 *μ*l of ATP detection working dilution were mixed for 3 s prior to the measurement of luminescence 10 s in a GloMax 20/20 luminometer (Promega, Madison, USA). All assays were performed in triplicate.

### Quantitative real-time PCR (qRT-PCR)

Quantitative real-time PCR (qRT-PCR) was performed to determine the PCV2 DNA copies as previously described.^55^ In brief, DNA was extracted using the TaKaRa DNA Mini kit (TaKaRa, China). The purified DNA was used as a template for PCR amplification. A pair of PCV2-specific primers (forward primer 5′-TAGTATTCAAAGGGCACAG-3′, reverse primer 5′-AAGGCTACCACAGTCAG-3′) was designed to amplify a 117-bp fragment from the PCV2 ORF2 gene. The qRT-PCR was performed using the ABI Prism Step One Plus detection system (Applied Biosystems, NY, USA). A recombinant pMD19 plasmid vector (TaKaRa) containing a PCV2 genome was used to construct a standard curve, and a TaKaRa SYBR green real-time PCR kit was used to measure the amount of viral DNA.

### Indirect immunofluorescence assay

PCV2-infected cells were detected using an indirect immunofluorescence assay (IFA) as previously described. PK-15 cells were washed three times with PBS containing 0.1% Tween-20 (PBST) and fixed in 4% paraformaldehyde for 20 min at room temperature (RT). After three washes in PBST, the cells were permeabilized with 0.1% Triton X-100 and then incubated in PBST containing 1% bovine serum albumin (BSA) for 1 h at 37 °C to block nonspecific binding. The cells were then incubated with porcine anti-PCV2 antibody (UnivBiotech, Shanghai, China) diluted in PBST containing 1% BSA (PBSTB; 1:50) for 1 h at 37 °C, and after three washes with PBST, FITC-conjugated rabbit anti-pig antibody (Sigma; diluted 1:100 in PBST) was added, and the cells were incubated for 1 h at 37 °C. The cells were again washed three times with PBST, and examined under a fluorescence microscope. Cells positive for PCV2 viral antigens were counted in six fields of view.

### Quantification of virus titer

PCV2-infected PK-15 cells grown in six-well plates were pretreated with DMSO or chemicals (3-MA or CQ) for 4 h, or transfected with ssiRNA, siATG5 or siBeclin-1 for 5 h. The cells were then treated with OTA for an additional 48 h. Samples of the culture supernatant samples were collected, and the cells were subjected to three freeze-thaw cycles. Total virus yield (intracellular and extracellular viruses) was determined by inoculating confluent PK-15 cells in 96-well culture plates with 10-fold dilutions of the culture supernatants. After 72 h of incubation, the viral antigen was detected by IFA as described above. Viral titers were calculated using the Reed-Muench method and expressed as TCID_50_/ml.

### Cell lysis and western blotting analysis

PK-15 cells cultured in six-well plates were scraped from the plates at the specific times indicated in the figures and collected in cell lysis buffer containing protease inhibitor (Beyotime, Haimen, China) on ice. The cell lysates were sonicated using a Sonics VCX105 sonicator and centrifuged at 12 000 rpm for 20 min at 4 °C. Protein concentration was determined using the BCA protein assay kit (Beyotime). Equal amounts of protein samples were diluted in 5 × SDS–PAGE loading buffer and heated at 95 °C for 5 min. The samples were separated on 12% SDS–PAGE gels and transferred to polyvinylidene fluoride membranes. After blocking for 1 h at RT in Tris-buffered saline containing 5% nonfat milk powder and 0.1% Tween 20, the membranes were incubated with primary antibodies overnight at 4 °C. The membranes were then washed and incubated in secondary antibody at RT for 1 h. Images of the immunoblots were acquired using an EU-88 image scanner (Seiko Epson Corp.). Quantification of protein blots was performed using the Image-Pro Plus 6.0 software (Media Cybernetics).

### Confocal fluorescence microscopy

Confocal fluorescence microscopy was used for the analysis of LC3 expression after OTA treatment. Specifically, PK-15 cells grown on coverslips to 40–50% confluence were transfected with the plasmid GFP-LC3 using X-tremeGENE HP DNA transfection reagent (Roche, Indianapolis, USA) according to the manufacturer’s guidelines. The cells were then infected with PCV2 for 24 h and inoculated with OTA for an additional 48 h as described above. The intracellular localization of LC3 was visualized using a Nikon C1-si confocal fluorescence microscope (Nikon Instruments, Inc.).

### RNA interference

ATG5-specific and Beclin-1-specific siRNAs were designed using the sequence of *Sus scrofa* ATG5 mRNA (GenBank Accession No. NM_001037152.1) and Beclin-1 mRNA (GenBank Accession No. NM_001044530.1), respectively, and Invitrogen BlockiT RNAi designer. Control siRNA sequences were obtained from published material.^63^ The ATG5-specific siRNA sequence was 5′-GCUUCGAGAUGUGUGGUUUtt-3′, the Beclin-1-specific siRNA sequence was 5′-CCUGGAUCGUGUUACCAUUtt-3′, and the sequence of control siRNA was 5′-UUCUCCGAACGUGUCACGUtt-3′. The three double-stranded RNAs were synthesized by Invitrogen. Duplex RNAs were resuspended in DEPC water to obtain 20 *μ*M solutions prior to use. PK-15 cells in DMEM 8% FBS without antibiotics were seeded in six-well plates at a density of 1 × 10^5^ cells/well and incubated for 24 h at 37 °C. When the cells were 30–50% confluent, siRNA was introduced using the X-treameGENE siRNA transfection agent (Roche) according to the protocol provided by the manufacturer. Transfection reagent (2.5 *μ*l) and 0.5 *μ*g siRNA were added to each well, and the plates were incubated for 5 h. The cells were then washed with DMEM and cultured in DMEM + 4% FBS until further treatments.

### Measurement of intracellular ROS

For the determination of ROS, PK-15 cells were cultured in six-well plates at a density of 1 × 10^5^ cells/well. Intracellular ROS levels were measured using 2′,7′-dichlorofluorescein diacetate (DCFH-DA; Beyotime) as previously described. Briefly, after removing the culture medium, the cells were incubated with DCFH-DA (10 *μ*M) at 37 °C for 30 min. The ROS level was determined by fluorescence microscopy and flow cytometry.

### Animal experiments

OTA extracts obtained from *in vitro* cultures of *Aspergillus ochraceus* strain (No. 3.4411) were added to the pig basal diet to provide the diets containing 70 and 150 *μ*g/kg OTA.

The experiment was conducted at a 300-sow pig farm. Sixty weaning piglets (age 6 weeks) were selected for the experiment. The average of PCV2 DNA copies in these animals, as measured by real-time PCR, was 10^3^–10^5^ due to natural infection. It was also determined by PCR that the piglets were negative for swine fever virus, swine influenza virus, porcine parvovirus, and porcine reproductive and respiratory syndrome virus. The experiments were conducted in accordance with the standards of the European Guidelines for Animal Welfare and were approved by the Committee for the Care and Use of Experimental Animals of the Nanjing Agricultural University (Animal Ethics Number: SYXK (Su) 2011-0036).

Twenty-seven piglets with an approximately equal body weights of 10.5 kg were selected, the average of PCV2 DNA copies in each of these animals measured by real-time PCR was 10^3^.37 The piglets were randomly divided into three groups, each group included three replicates, with 3 piglets per replicate. Group I, the control group, received a basal diet, groups II and III received the basal diet containing 75 and 150 *μ*g/kg OTA, respectively, for 42 days. The piglets were housed in different rooms under biosafety conditions with *ad libitum* access to water and feed during the experiment. At the end of the experiment, the piglets were killed, and tissue samples of liver, kidney, spleen, lung, ILN, and BLN were collected. The animal’s serum was analyzed for SOD activity and MDA content. Tissue samples were analyzed for OTA concentration and PCV2 DNA copies and the proteins were extracted for western blotting analysis.

### Determination of OTA concentration by LC-MS/MS

OTA was extracted from tissues using the method as previously described. Briefly, 1 ml of tissue homogenates was mixed with 5 ml of a methanol/water (80/20) mixture. After vortexing for 2 min, the mixture was sonicated for 1 h, and centrifuged for 15 min at 3300 × *g*. The supernatant fluid was transferred to a 10-ml centrifuge tube and dried under nitrogen gas at 50 °C. The residue was reconstituted in 500 *μ*l acetonitrile/water (20/80, v/v) containing 10 mmol/l ammounium acetate, and the resulting solution was passed through a nylon membrance (0.22 *μ*m) prior to LC-MS/MS.

One milligram of OTA was dissolved in 5 ml of ethanol to yield a stock solution of 0.5 mg/ml OTA, which was stored at −20 °C in the dark. Standard solutions of OTA were prepared by diluting the stock solution with a mixture of acetonitrile/water (20/80, v/v) containing 10 mmol/l ammonium acetate. A commercially available stock solution of the IS([13C20]-OTA) was diluted with the same solution to 50 ng/ml. All working solutions were prepared immediately before use.

LC-MS/MS (TSQ Quantum Ultra, Thermo Scientific, USA) equipped with electro-spray ionization was used for the analysis of OTA, as previously described.^7^

### Determination of SOD and MDA levels

The levels of SOD and MDA were detected using specific detection kits obtained from the Nanjing Jiancheng Institute of Biotechnology (Jiancheng, Nanjing, China) according to the manufacturer’s instructions. Serum SOD levels are expressed in U/ml, and MDA levels are expressed in nmol/ml.

### Statistical analysis

Statistical analyses were performed using the SPSS computer program for Windows (version 22.0). Data were analyzed for establishing their significance using one-way analysis of variance followed by least-significant difference test. Data are expressed as the mean±standard error (S.E.). Differences were regarded as significant at *P*<0.05.
